# Inflammatory gene expression profiling in peripheral blood from patients with Alzheimer’s disease reveals key pathways and hub genes with potential diagnostic utility: a preliminary study

**DOI:** 10.7717/peerj.12016

**Published:** 2021-08-20

**Authors:** Kelly Cardona, Javier Medina, Mary Orrego-Cardozo, Francia Restrepo de Mejía, Xabier Elcoroaristizabal, Carlos Andrés Naranjo Galvis

**Affiliations:** 1 Facultad de Salud, Universidad Autónoma de Manizales, Manizales, Caldas, Colombia; 2 Genetracer Biotech, Madrid, España

**Keywords:** Late-onset Alzheimer disease, RNA Ion AmpliSeq, Inflammation, Gene expression analysis, Hub genes

## Abstract

**Background:**

Alzheimer’s disease (AD) is an age-related neurodegenerative disease caused by central nervous system disorders. Late-onset Alzheimer disease (LOAD) is the most common neurodegenerative disorder worldwide. Differences at the expression level of certain genes, resulting from either genetic variations or environmental interactions, might be one of the mechanisms underlying differential risks for developing AD. Peripheral blood genome transcriptional profiling may provide a powerful and minimally invasive tool for the identification of novel targets beyond A*β* and tau for AD research.

**Methods:**

This preliminary study explores molecular pathogenesis of LOAD-related inflammation through next generation sequencing, to assess RNA expression profiles in peripheral blood from five patients with LOAD and 10 healthy controls.

**Results:**

The analysis of RNA expression profiles revealed 94 genes up-regulated and 147 down-regulated. Gene function analysis, including Gene Ontology (GO) and KOBAS-Kyoto Encyclopedia of DEGs and Genomes (KEGG) pathways indicated upregulation of interferon family (INF) signaling, while the down-regulated genes were mainly associated with the cell cycle process. KEGG metabolic pathways mapping showed gene expression alterations in the signaling pathways of JAK/STAT, chemokines, MAP kinases and Alzheimer disease. The results of this preliminary study provided not only a comprehensive picture of gene expression, but also the key processes associated with pathology for the regulation of neuroinflammation, to improve the current mechanisms to treat LOAD.

## Introducion

Alzheimer’s disease (AD) is the most common neurodegenerative disorder worldwide. Is the most common cause of dementia, accounting for an estimated 60% to 80% of cases (Alzheimer’s Association, 2021). The pathophysiological mechanism of AD remains unclear, the etiology of the disease is multifactorial; its progression involves multiple physiological, environmental and genetic factors (Bagyinszky et al., 2014). After age, sex is one of the strongest risk factors for developing AD, Currently, over 65% of people with late-onset Alzheimer’s Disease (LOAD) are women (Alzheimer’s Association, 2019). In Alzheimer’s disease (AD), neuroinflammation, instead of being a mere bystander activated by emerging senile plaques and neurofibrillary tangles, contributes as much or more to the pathogenesis as do the plaques and tangles themselves. Inflammation is a complex molecular and cellular defense mechanism in response to diverse stimuli, including stress, injury, and infection. Inflammation has been well established as a major component of neurodegenerative disorders, but it has never been clear if this was a direct cause of the disease or a consequence of the progressive degenerative process that was occurring (Zhang et al., 2013). In the brain, an upregulation in inflammatory signaling is characterized by the activation of astrocytes and microglia and pro-inflammatory mediators (Liu & Chan, 2014; Martorell et al., 2013). In the last decades, the molecular level of human diseases has been challenging to approach and has been explored mostly with different technologies such as high-throughput sequencing. There is the increasing opportunity to link large global datasets with the technologies of genomics, proteomics and transcriptomics through bioinformatics methods to unlock the genetic, molecular pathways and physiological that underpin the pro-inflammatory aging-phenotype. This approach takes on special importance in the case of inaccessibility and invasiveness of obtaining samples from certain organs such as the brain- hindering the analysis of neurological diseases. Peripheral blood samples are possible subrogates of pathways triggered in the brain, to reflecting some of the same expression evolution and regulation (Cai et al., 2010; Griswold et al., 2020). Blood is one of the most clinically convenient tissues to sample for immune biomarkers, whereas the brain is arguably the least convenient. Circulating blood is easily accessible and can represent an alternative to tissue sampling to find molecular signatures of different physiological conditions. Blood-based biomarkers have been suggested to be useful for evaluating diagnosis, pathogenesis, and AD progression. A recent study of differential expression in AD from different tissues shows that many genes differentially expressed in AD were regulated similarly in blood and brain (Bai et al., 2014). For blood biomarkers to be relevant to the inflammatory pathogenesis of AD, it is assumed that the immune status of the central nervous system is correlated with, or caused by, the immune status of the periphery. Chronic inflammation leads to various diseases such as cardiovascular diseases, cancer, inflammatory bowel disease and Alzheimer’s disease, etc., due to the involvement of numerous inflammatory pathways such as TNF-*α*, NF-*κ*B, STAT, MAPK and JNK (Chen et al., 2018). In our study, we used female LOAD and Healthy Control Samples to obtain information about systemic factors that might influence Alzheimer’s disease using RNA Ion AmpliSeq sequencing. The data reported herein represent changes AD patients’ immune cells and supports that LOAD physiopathology relates to inflammatory cells as a hypothesis. Gene function analysis, including Gene Ontology (GO) and KOBAS-Kyoto Encyclopedia of DEGs and Genomes (KEGG) pathways analysis, were performed using differentially expressed genes (DEG) on Metascape (https://metascape.org/gp/). The present study characterizes intrinsic molecular processes underlying LOAD and our preliminary data suggests that we may have identified possible biomarkers for AD.

## Materials & Methods

This study was conducted using peripheral blood samples from 15 female participants over 70 years old diagnosed with LOAD (5) and demographically matched healthy controls (10). Alzheimer’s Disease has a greater incidence and is more prevalent in women as compared with men (Laws, Irvine & Gale, 2016). Besides, evidence indicates that the APOE-*ɛ*4 risk for AD is greater in women than men, which is particularly evident in heterozygous women carrying one APOE-*ɛ*4 allele (Riedel, Thompson & Brinton, 2016). In addition, a previous study from our laboratory found some gender differences in the clinical course of Alzheimer’s disease in our population (Naranjo-Galvis et al., 2020, unpublished data). Women have a higher survival rate but more disability. For example, having an apolipoprotein E *ɛ*4 allele (+APOE-*ɛ*4) places women at a higher risk of developing AD, compared to men. All women included in this study have an apolipoprotein E*ɛ*4 allele (+APOE-*ɛ*4) (Naranjo-Galvis et al., 2020, unpublished data). Control participants were invited through an open call, while subjects with LOAD were referred by neurology specialists, only those subjects diagnosed with LOAD according to the GDS (Reisberg et al., 1982) and CDR criteria (Morris, 1993), were recruited for the study. Subjects with a history of psychiatric or neurological disorders that explain their cognitive or behavioral changes, severe auditory or visual deficits, history of substance abuse, allergic illness, systemic inflammatory disease or unstable medical conditions, or those who were consumed systemic corticosteroids at least one month before the test were excluded from the study. The participants and LOAD subject’s guardian signed a written informed consent approved by the Ethics Committee of the Universidad Autónoma de Manizales (Act 047 June 2015), after the procedure’s totality was explained to them (Project 02110402025). Participants provided a sociodemographic and pathological history questionnaire and the mini-mental state examination (MMSE) (Folstein, Folstein & McHugh, 1975). Later, 5 ml of peripheral blood was obtained in accordance with the Colombia Ministry of Health’s biosafety regulations and guidelines.

Initial RNA extraction was performed using the Tempus™ Spin RNA Isolation Kit (Ref. 4380204; Thermo fisher Scientific, Waltham, MA, USA) following the manufacturer’s recommendations. The total RNA obtained was quantified using the Agilent RNA 6000 Pico microfluidics chip (reference 5067-1513) in the Bioanalyzer 2100 equipment (Agilent Technologies, Palo Alto, CA, USA) following the manufacturer’s instructions.

Transcriptome sequencing was performed using the Ion AmpliSeq Transcriptome Gene Expression Kit (Thermo Fisher Scientific). The library was prepared on 40 ng of total RNA using the AmpliSeq Transcriptome Kit v2 (reference 4475936; Thermo Fisher Scientific) combined with barcodes Ion Xpress™ 01-16 Kit (4471250AB, Thermo Fisher Scientific) following the manufacturer’s specifications. The resulting libraries were quantified with the Ion Library TaqMan™ Quantification Kit (Ref. 4468802; Thermo Fisher Scientific) in a final volume of 10 uL. The enrichment of the carried ISPs and the loading of the chip were developed in the automated Ion Chef system (Thermo Fisher Scientific) using the Ion PI™ Hi-Q™ Chef Kit (reference A27198; Thermo Fisher Scientific) and the Ion PI™ chips v3 (reference A26771; Thermo Fisher Scientific). Then, the chip was analyzed in the Ion Proton sequencer (reference 4476610; Thermo Fisher Scientific), and the data generated were initially analyzed with the Torrent Suite 5.0.2 to generate the sequence of reads, eliminate bar codes, and low-quality reads. With the resulting reads (>80 million), FASTQ files were generated using the File Exporter 4.6.0.0 plugin.

AmpliSeq sequencing data were analyzed and adjusted by quality using SAMStat v 1.5 (Lassmann, Hayashizaki & Daub, 2011) with the resultant data checked for quality using FastQC. The data were aligned to the human reference genome annotation UCSC Hg19 using the Ion Torrent Mapping Alignment Program (TMAP).

RNA expression values were determined with BED Tools v 2.26 (Quinlan, 2014). and differentially expressed genes (DEGs) were obtained using the R package DeSeq2 v.1.12.4, available from Bioconductor (Love, Huber & Anders, 2014). Transcripts were excluded from any subsequent analysis if one point in the pairwise analysis did not have at least 50 reads across all the samples. Besides, a criterion of at least three replicates per condition that would support a differentially expressed gene was applied to select DEG. The false discovery rate (FDR) was set to a cut-off of 0.05 for all transcripts. Specifically, we applied the Bonferroni correction for paired comparisons, and only accepted genes with corrected significance (*P* value) < 0.05.

DEG was converted to their corresponding Homo sapiens Entrez gene IDs using the latest database (ncbi.nlm.nih.gov/gene; updated on 2020-09-15); pathway and process enrichment analysis of DEG was performed using Metascape (metascape.org) (Zhou et al., 2019). For each given gene list, pathway and process enrichment analysis has been carried out with the following ontology sources: GO Biological Processes, KEGG Pathway, Canonical Pathways, Reactome Gene Sets, TRRUST, CORUM, DisGeNET, PaGenBase, Transcription Factor Targets and COVID.

All genes in the genome were used as an enrichment background. Terms with *P* < 0.01 were statistically significant. Those with a minimum count of three and enrichment factor >1.5 were collected and grouped into clusters based on their membership similarities. More specifically, *P*-values were calculated based on accumulative hypergeometric distribution (Feinleib & Zar, 2006), and q-values were calculated using the Benjamini-Hochberg procedure for accounting for multiple testing (Hochberg & Benjamini, 1990). Kappa scores were used as the similarity metric when performing hierarchical clustering on the enriched terms, and then sub-trees with similarity > 0.3 were considered a cluster (Cohen, 1960). The most statistically significant term within a cluster was selected to represent the cluster. For each given gene list, protein-protein interaction enrichment analysis was carried out with the following databases: BioGrid (Stark, 2005), InWeb_IM (Li et al., 2016) and, OmniPath (Türei, Korcsmáros & Saez-Rodriguez, 2016). The resultant network contains the subset of proteins that form physical interactions with at least one other member in the list.

Convergent functional genomic (CFG) is a methodology that integrates multiple lines of external evidence (Genetic association of DNA variations with disease susceptibility; gene expression regulated by AD genetic variants; protein-protein interaction with AD core proteins, *i.e.,* APP, PSEN1, PSEN2, APOE and MAPT; diagnosis prediction of disease models) from animal and human model studies (Niculescu & Le-Niculescu, 2010) aiming at prioritizing AD candidate genes (Xu et al., 2018). The DEGs were scored based the CFG score of each gene ranged from 0 to 6 points supported by the above-mentioned evidence from the AlzData database (CFG score); the higher the score, the more promising the gene is.

## Results

RNA was isolated from whole blood from 5 LOAD cases and 10 controls (all female. Mean age 76.3 ± 3.5). The amplicon sequencing approach using 20,812 probes sets specific to human mRNA was applied to assess gene expression profiles, yielding 12.3 million reads per sample with alignment rates higher than 94.7%.

**Figure 1:**
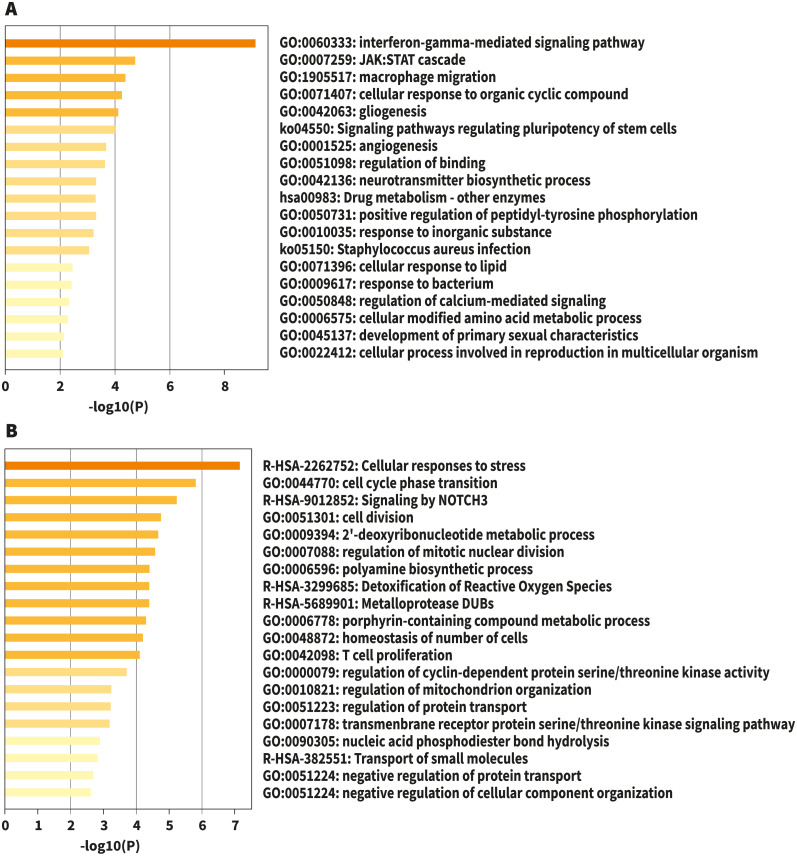
Bar graph of enriched terms across input gene lists, colored by *p*-values. (A) Upregulated. (B) Downregulated. Image credit: Metascape.org.

**Figure 2:**
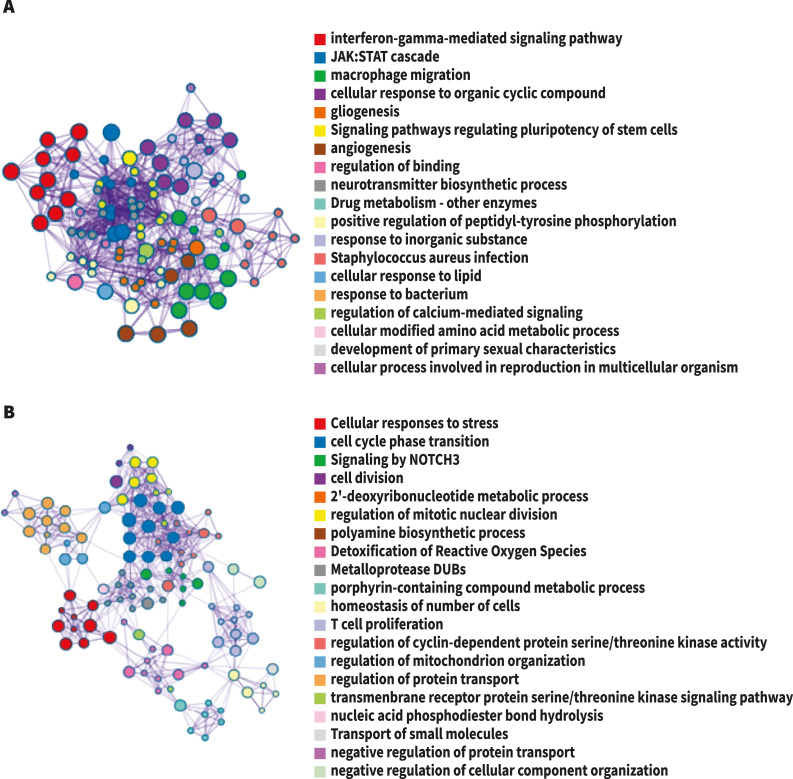
The network of enriched terms colored by cluster-ID. Nodes sharing the same cluster-ID are typically close to each other, terms containing more genes tend to have a more significant *p*-value. (A) Up-regulated DEG. (B) Down-regulated DEG. Image credit: Metascape.org.

For analysis of differential gene expression between LOAD cases and controls, signals from 13,227 amplicons with mean mapped reads higher than fifty reads across all participants were included. Differential gene expression analysis revealed 241 DEG (FDR<0.1): 94 up-regulated, and 147 down-regulated considering values of *P* < 0.05 (Table S1). Functional enrichment analyses of the DEG were carried out through gene ontology (GO) (Ashburner et al., 2000). To further capture the relationships between the terms, a subset of enriched terms has been selected and displayed as a network plot, where edges connect terms with a similarity >0.3. We selected the terms with the best *p*-values from each of the 20 clusters, with the restriction that there are no more than 15 terms per cluster and no more than 250 terms in total (Fig. 1). The network visualization was performed using Cytoscape (Shannon et al., 2003), where each node represents an enriched term and has been colored by its cluster-ID (Fig. 2).

The resultant network contains the subset of proteins that form physical interactions with at least one other member in the list. If the network contains between three and 500 proteins, the Molecular Complex Detection (MCODE) algorithm has been applied to the resultant network to identify densely connected network components (Bader & Hogue, 2003). Pathway and process enrichment analysis has been applied to each MCODE component independently, and the three best-scoring terms by *p*-value have been retained as the functional description of the corresponding components (Table 1).

**Figure 3:**
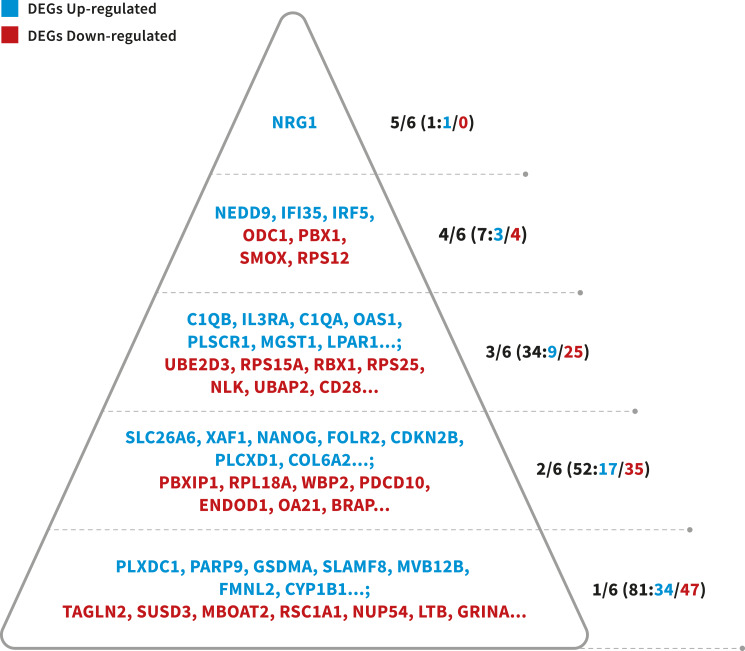
Probability pyramid representing the results of gene prioritization for the 175 DEGs (64 Up-regulated, 111 Down-regulated). The highest CFG score is 6. Colors represent different genes in our study. Blue represents the DEGs Up-regulated, whereas read means the DEGs Down-regulated. Image credit: the authors.

To identify more reliable candidate genes from a large number of LOAD-related genes, a prioritized list of the DEGs was generated using a convergent functional genomics (CFG) method (Table S1). For the 241 candidate genes (241 DEGs), 175 genes (64 up-regulated and 111 down-regulated) were listed as highly LOAD-related candidate genes when received at least two lines of LOAD-related evidence (CFG score > 1, Fig. 3). The interesting thing is that these genes together play a very important role in the immune response. In summary, this study we have also identified important genes (NRG1, NEDD9, IRF5, IFI35, STAT1, STAT2, JAK2, IL3RA), and alterations in biological processes and pathways that may be associated with Alzheimer’s Disease. Our study may provide effective means for the prevention, diagnosis, and treatment Alzheimer’s Disease in female patients.

**Table 1:** GO enrichment analysis was applied to each MCODE network to assign “meanings” to the network component, where the top three best *p*-value terms were retained.

**Category DEGs**	**GO**	**Description**	**LogP**
Upregulated Upregulated Upregulated	GO:0097696	STAT cascade	−6.4
	GO:0007259	JAK-STAT cascade	−6.5
	GO: 0042063	Gliogenesis	−6.6
Downregulated	R-HSA-9010553	Regulation of expression of SLITs and ROBOs	−6.8
Downregulated	R-HSA-8953897	Cellular responses to external stimuli	−7.9
Downregulated	R-HSA-2262752	Cellular responses to stress	−8.0

Even when this study is the first study carried out in Alzheimer’s Disease sufferers of this part of Colombia, the sample size of the affected included is limited. The homogeneity in the symptoms of the patients selected was determinant for their inclusion together the fact that all should belong to the same region. Despite these matters, the extrapolation of our main results must be considered carefully for the whole population of the region and therefore understand as a limitation. Nonetheless, a follow-up study later for a bigger size is required for future confirmations.

## Discussion

The availability of an accessible tissue where gene expression profile is similar to more inaccessible CNS tissues has the potential to advance research in neuropsychiatric disorders, and various studies have suggested that on a transcriptome level, whole blood shares significant gene expression similarities with multiple CNS tissues (Sullivan, Fan & Perou, 2006; Leandro et al., 2018; Griswold et al., 2020; Shigemizu et al., 2020). Furthermore, it is now widely accepted that inflammation plays an important role in the pathogenesis of AD (Heppner, Ransohoff & Becher, 2015). In this way, while prominent neuroinflammation occurs in the brain parenchyma during LOAD, we also observed a peripheral inflammation, including the active involvement of peripheral immune cells such as T lymphocytes or monocytes (Mietelska-Porowska & Wojda, 2017).

Our study with five LOAD cases and 10 healthy controls demonstrates differences in gene expression profiles between both samples. Measuring gene expression using the AmpliSeq approach, a method of high sensitivity and reproducibility (Li et al., 2015), facilitated the detection of 241 genes differentially expressed in LOAD even with a small sample size. Although samples were from blood and not from CNS cells directly. However, the identified genes could be relevant in LOAD’s physiopathology. While some studies of differential expression in LOAD have been carried out in postmortem brains (Neff et al., 2021), other research (Leandro et al., 2018; Griswold et al., 2020; Shigemizu et al., 2020) and ours address the study of LOAD mechanisms through a non-invasive perspective. Being able to evaluate LOAD *in vivo* is opening future perspectives for identifying pathways contributing to the early diagnosis of the disease.

Up-regulated genes were enriched in interferon-gamma (INF-*γ*) pathways. INF-*γ* can activate a cytotoxic response in the brain; high cytokine levels impact astrocytes’ activation, which induces neurotoxicity and cell death. Experimental evidence has shown that stimulation with INF-*γ* increases more than four times the percentage of cell death because astrocytes increase their neurotoxicity and, therefore, their potency to kill neuronal tissue (Hashioka et al., 2009). The overexpression of genes related to INF-*γ* induces both gene expression and enzymatic activity of indoleamine 2,3-dioxygenase (IDO-1), an enzyme expressed in numerous cell types, including macrophages, microglia, neurons, and astrocytes. This enzyme is fundamental in the kynurenine pathway, which plays a significant role in the pathogenesis of a wide variety of inflammatory, neurodegenerative, and malignant disorders (Jones et al., 2015).

Furthermore, INF-*γ* is an activator of the JAK/STAT pathway that modulates the transcription of different inflammatory factors (Platanias, 2005). In our research, four genes involved in the JAK/STAT pathway were up-regulated (STAT1, STAT2, JAK2, IL3RA). Biochemical studies of JAK2 have established their participation in the signaling of different cytokines, including IL-3 receptors (Schindler & Strehlow, 1999); JAK2 has a critical role in the transduction of IL-3, which plays an important role in cell growth and differentiation (Testa, Pelosi & Castelli, 2019). Studies in rats suggest that IL-3 has a potential role in inflammatory processes in NCS, *in vitro* studies suggest that IL-3 could mediate microgliosis and neurodegeneration in LOAD. Likewise, the increase in the expression of IL-3R *α* is associated with tumorigenesis (Lee, Martin & Merrill, 1993).

So, we hypothesized that the alteration in the JAK / STAT pathway due to the overexpression of the STAT1, STAT2, JAK2, and IL3R genes has two different mechanisms one: dysregulation of JAK2 and IL3R that result in the alteration of the production of the cytokine IL-3 and the appearance of reactive microgliosis and subsequent neurodegeneration in the brain, very common in LOAD, and the second one, the deregulation of STAT1 and STAT2 that together with JAK2 are involved in the activation of the transcription of multiple cytokines of the INF family, which can result in a chronic pro-inflammatory response, alterations in cell cycle regulation and apoptosis, So, it is possible that neurotoxic effect of astrocytes derived from INF-*γ* could be reversed by using JAK/STAT pathway inhibitors (Hashioka et al., 2009) which makes the JAK/STAT pathway a potential therapeutic target against LOAD and other related neurodegenerative diseases. Compared to our study, Morabito et al. (2020) revealed specific transcriptomic signatures across different brain datasets. Several pathways were upregulated, such as mitogen-activated protein kinase (MAPK) signaling and Janus kinase (JAK)-signal transducer/activator of transcription (JAK/STAT) involved in numerous cellular processes and it is implicated in neurodegenerative disorders, like Alzheimer disease. Additionally, we validate our results in multiple published human AD gene expression datasets, which can be easily accessed using online resource (https://swaruplab.bio.uci.edu/consensusAD) in order to provide a more comprehensive picture of the genome-wide transcriptomic alterations occurring in AD and to begin to examine the associated gene regulatory programs (Morabito et al., 2020). A better understanding of inflammatory response pathways and molecular mechanisms will undoubtedly contribute to improved prevention and treatment of inflammatory diseases.

**Figure 4:**
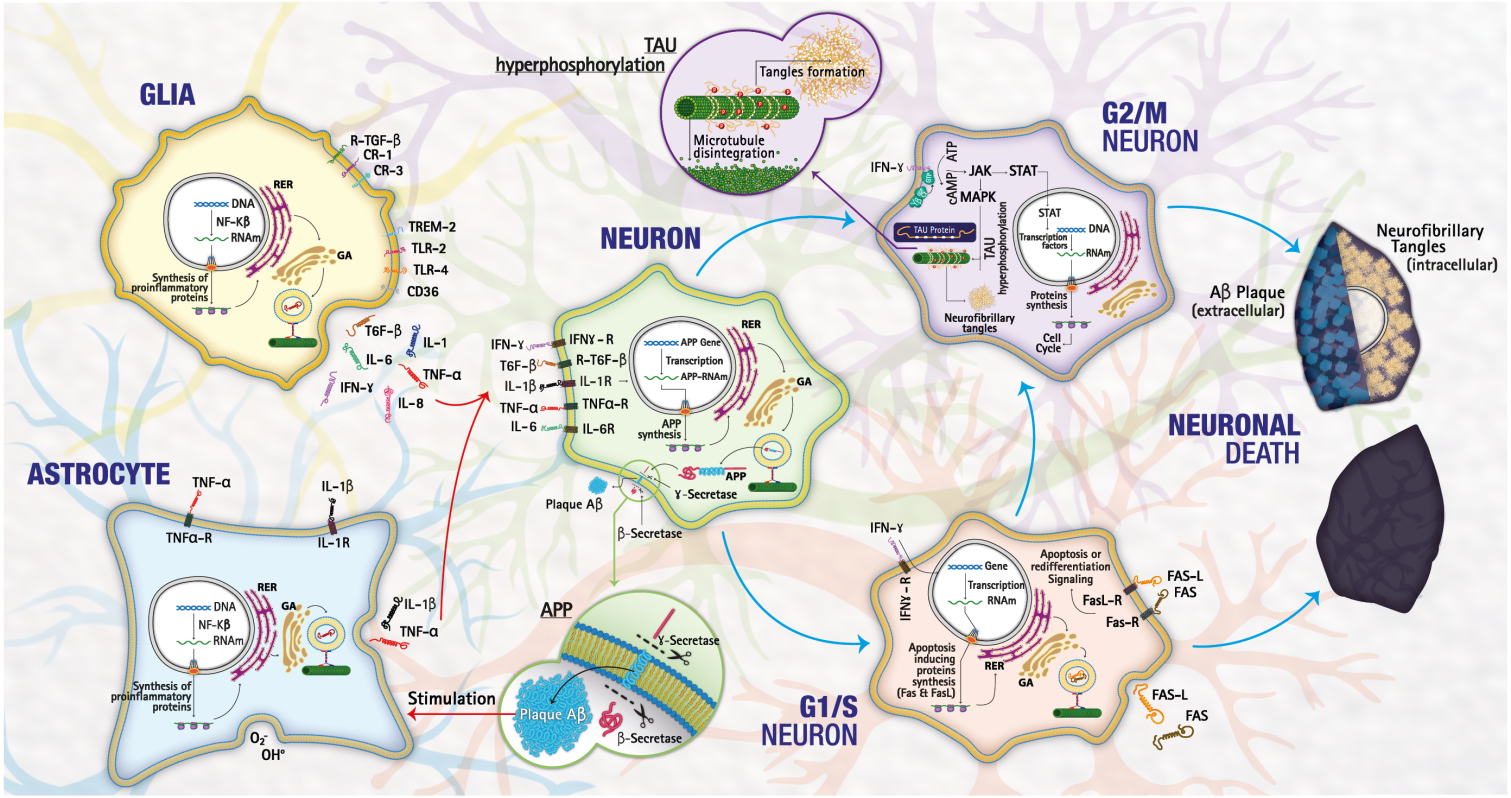
Mechanisms of cell death. The lower pathway shows a normal process of death by necrosis/apoptosis, the upper pathway a death by neurodegeneration as happens in LOAD. Image credit: the authors.

From the results obtained in the functional enrichment analysis numerous downregulated genes were involved in biological processes associated with the cell cycle; allowing us to hypothesize a link between the cell cycle dysregulation and the inflammation in LOAD. It is well known that in the adult brain, the neurons are in phase G0; these are not dividing and are terminally differentiated. Therefore, the re-expression of cell cycle proteins is considered a pathological phenomenon because the deregulation of the genes involved in G1/S and G2/M allows the neuron to escape from apoptosis and initiate a continuous degenerative process (Hamdane et al., 2003), triggering tauopathies as observed in LOAD (Tatebayashi et al., 2006). Besides, , the re-expression of G1/S markers correlates with apoptosis in many tissues (Li et al., 2015). Inflammation in the CNS occurs mainly through the activation of astrocytes and microglia and the release of numerous factors like chemokines, cytokines, or growth factors, leading to a complex crosstalk between different brain cell types (astrocytes, microglia, endothelial cells, neurons, *etc.*). Among cytokines, IFN-*γ* is an important activator of the JAK-STAT signaling pathway (Regis et al., 2008). CNS dysregulation of the JAK-STAT pathway is mainly related to brain inflammation processes and neuronal/glial survival. In particular, STAT proteins can either promote cell death and contribute to brain damage (Fig. 4).

We investigated DEG genes with high CFG scores for their correlation to AD. Among 175 DEGs, NRG1 showed a CFG score of five points. Studies recently explain the relation between the pivotal role of NRG-1 and inflammation in the development of the cognitive disorders. NRG-1 gene plays an important role in plasticity, maintenance and repair of the nervous system. Moreover, NRG-1 has been found in multiple regions of the adult brain and is involved in the regulation of neurotransmission (Mostaid et al., 2017). Due to its localization in the brain areas relating to auditory and visual hallucinations and delusions, it has been hypothesized that the presence of NRG-1 may interact with specific inflammatory pathological processes that have been shown to be associated with Late Onset Alzheimer’s Dementia (LOAD) (Go et al., 2005).

Thus, because LOAD behaves as a multifactorial entity, the formulation of new etiopathogenic models, which include new contributions emerging from areas such as neuroimmunogenetics, may be informative. Despite the mechanisms of the observed blood–brain and blood-tissue links are not completely established this approach is valuable because facilitates the study of LOAD mechanisms through a less invasive perspective, the fact of being able to evaluate LOAD *in vivo* expands the perspectives for identifying biological factors contributing to the early diagnosis, prognosis and treatment of the disease.

These are promising data—considering the peculiarities of our study—but overall, the limitations of the list of genes identified as differentially expressed must be understood. Mostly those genes that have a greater difference between the samples analyzed will have been identified while those for which there is a smaller difference between groups / or a big variance will probably not have been identified.

## Study Limitations

There are some limitations in our approach. First, as a new method for sequence-based, genome-scale gene expression quantification, AmpliSeq stands as a very versatile and cost-effective approach for gene expression analysis with high accuracy (Li et al., 2015). However, to ensure sufficient statistical power, studies of functional consequences of differential gene expression generally entail a large scale of cohorts. Due to the small size sample in our research further validation of our findings is required and additional investigation in larger populations is necessary to find out clinical implications. Second, gene expression between brain and peripheral blood are not completely correlated (Griswold et al., 2020) and thus brain specific pathways will not be represented here. In addition, whole blood represents a heterogeneous mixture of cell types (*e.g.*, monocytes, neutrophils, B-cells, T-cells *etc.*) which could introduce a bias in the results due to the proportions of these cell types could differ by unknown covariates and cell specific effects could be masked. However, our study’s strength is the appropriate adjustment of variables such as age and sex and the stringent selection criteria of patient samples.

## Conclusions

Immune function is an important mechanism in LOAD pathogenesis and differential expression of immune genes has been noted in studies of AD brain and consistent in blood (Ma et al., 2019; Stopa et al., 2018) being remarkable the role of interferon response. Blood is one of the most clinically convenient tissues to sample for immune biomarkers, whereas the brain is arguably the least convenient. For blood biomarkers to be relevant to the inflammatory pathogenesis of Alzheimer’s disease, it is assumed that the immune status of the central nervous system is correlated with, or caused by, the immune status of the periphery. The findings of AD-related genes from blood samples in a larger study could provide biomarkers of AD’s progression and potential therapeutic targets to the development of AD diagnostic and treatment tools.

##  Supplemental Information

10.7717/peerj.12016/supp-1 Supplemental Information 1 Differential gene expression analysis 241 DEG (FDR <0.1): 94 up-regulated, and 147 down-regulated considering values of *P* < 0.05.

## Additional Information and Declarations

### Data Availability

The following information was supplied regarding data availability: Data is available at NCBI GEO: GSE168813.
